# Nonsymbolic and Symbolic Numerical Magnitude Processing in the Brazilian Children with Mathematics Difficulties

**DOI:** 10.1590/1980-57642021dn15-040013

**Published:** 2021

**Authors:** Isabella Starling-Alves, Annelise Júlio-Costa, Ricardo José de Moura, Vitor Geraldi Haase

**Affiliations:** 1 Educational Psychology Department, University of Wisconsin-Madison – Madison, WI, USA.; 2 Developmental Neuropsychology Lab, Universidade Federal de Minas Gerais – Belo Horizonte, MG, Brazil.; 3 Basic Psychology Processes Department, Instituto de Psicologia, Universidade de Brasília – Brasília, DF, Brazil.; 4 Department of Psychology, Universidade Federal de Minas Gerais – Belo Horizonte, MG, Brazil.; 5 raduation Program in Psychology: Cognition and Behavior, Universidade Federal de Minas Gerais – Belo Horizonte, MG, Brazil.; 6 National Institute of Science and Technology: Behavior, Cognition, and Instruction, Universidade Federal de São Carlos – São Carlos, SP, Brazil.

**Keywords:** mathematics, neuropsychology, dyscalculia, matemática, neuropsicologia, discalculia

## Abstract

It is still debated if the main deficit in mathematical difficulties (MD) is nonsymbolic or symbolic numerical magnitude processing.

**Objectives:**

In the present study, our main goal was to investigate nonsymbolic and symbolic numerical magnitude processing in MD and the relationship between these abilities and arithmetic.

**Methods:**

The Brazilian school-age children with MD completed a nonsymbolic and a symbolic numerical magnitude comparison task and an arithmetic task. We compared their performance with a group of children with typical achievement (TA) and investigated the association between numerical magnitude processing and arithmetic with a series of regression analyses.

**Results:**

Results indicated that children with MD had low performance in the nonsymbolic numerical magnitude comparison task. Performance in both nonsymbolic and symbolic numerical magnitude comparison tasks predicted arithmetic abilities in children with TA, but not in children with MD.

**Conclusions:**

These results indicate that children with MD have difficulties in nonsymbolic numerical magnitude processing, and do not engage basic numerical magnitude representations to solve arithmetic.

## RESUMO

Ainda é debatido se o principal déficit nas dificuldades matemáticas (DM) se dá no processamento de magnitudes numéricas não simbólicas ou simbólicas.

**Objetivos:**

No presente estudo, nosso principal objetivo foi investigar o processamento não simbólico e simbólico de magnitudes numéricas na DM e a relação entre essas habilidades e a aritmética.

**Métodos:**

Crianças brasileiras em idade escolar com DM completaram tarefas de comparação de magnitudes numéricas não simbólicas e simbólicas e uma tarefa aritmética. Comparamos seu desempenho com o de um grupo de crianças com desempenho típico (TA) e investigamos a associação entre o processamento de magnitude numérica e a aritmética em uma série de análises de regressão.

**Resultados:**

Os resultados indicaram baixo desempenho na tarefa de comparação de magnitudes numéricas não simbólicas nas crianças com DM. Além disso, o desempenho nas tarefas de comparação de magnitudes numéricas não simbólicas e simbólicas foi preditor de habilidades aritméticas de crianças com TA, mas não de crianças com DM.

**Conclusões:**

Esses resultados indicam que crianças com DM têm dificuldades no processamento de magnitudes numéricas não simbólicas e não empregam representações básicas de magnitudes numéricas para resolver problemas aritméticos.

## INTRODUÇÃO

Mathematics difficulties (MD) impact children’s academic performance and social well-being.^1,2^ Children with MD are unsuccessful in performing addition, subtraction, and multiplications problems.^3,4,5^ Such struggle is usually manifested by high reaction times (RT), low accuracy, and the use of immature strategies, such as finger counting, while solving arithmetic operations.^3,6,7,8^

It is still unclear how basic number systems contribute to the difficulties observed in MD. Some numerical cognition models propose that complex mathematics skills build on primitive systems dedicated to processing nonsymbolic numerical magnitudes.^9^ Therefore, deficits in processing nonsymbolic numerical magnitude could lead to low arithmetic performance.^10^ On the contrary, other models propose that understanding symbolic numerical magnitudes is crucial to develop advanced mathematics knowledge and to refine our primitive, approximate numerical representations.^11^ The numerical cognition research community has debated whether children with MD have difficulties understanding nonsymbolic or symbolic numerical magnitudes.^10,11,12 13^

Several studies have shown that children with MD have low performance in nonsymbolic numerical magnitude comparison tasks.^14,15,16^ The ability to compare different numerical magnitudes depends on the ratio between them, with higher accuracy for increasing ratios, as stated by Weber’s law, and indexed by the Weber fraction.^17^ The Weber fraction accounts for the variability in the representation of a specific numerical magnitude, and the higher its value, the less accurate the sensitivity to numerical differences is.^17,18,19^ Several studies have found that children with MD have a higher Weber fraction — which is indicative of worse performance — than children with typical achievement (TA).^13,15,20,21^

In contrast with results showing nonymbolic numerical magnitude deficits in MD, other studies have failed to find such a pattern. Instead, some studies have found that children with MD have low performance in tasks assessing symbolic numerical magnitudes. More specifically, children with MD have been outperformed by children with TA in symbolic numerical magnitude comparison tasks that used single or multi-digit numbers as stimuli.^3,12,22,23^ In a literature review, Noël and Rouselle^11^ have found a systematic pattern of difficulties with symbolic numerical magnitude tasks in children with MD. However, difficulties with nonsymbolic numerical magnitude tasks were nonsystematic. These results suggest that the main deficit in MD is in processing symbolic — and not nonsymbolic — numerical magnitudes.

Despite several studies investigating whether children with MD have deficits in processing nonsymbolic or symbolic numerical magnitudes, this debate is still unsolved. One limitation in solving this debate is that inconsistent methods have been used in the literature, making it challenging to compare results across studies. Traditionally, studies that found that children with MD have deficits in comparing nonsymbolic numerical magnitudes used the Weber fraction as a performance index.^10,13,15^ In contrast, most studies that found that children with MD have deficits in comparing symbolic numerical magnitudes used either reaction time or accuracy.^22,24^ Furthermore, given the difficulties in conducting studies with special populations, few studies have analyzed nonsymbolic and symbolic number processing within the same group of children with MD.^3,14,21,22,23 25,26,27^

In the present study, we compared the performance of the Brazilian children with MD and TA in a nonsymbolic and a symbolic numerical magnitude comparison task. If the hypothesis that the main deficit in MD is processing nonsymbolic numerical magnitude is accurate, we may observe group differences in the nonsymbolic numerical magnitude comparison task. Otherwise, if the hypothesis that the main deficit in MD is processing symbolic numerical magnitude is accurate, we may observe group differences in the symbolic numerical magnitude comparison task. To reduce methodological issues, we computed the Weber fraction as an index of performance in both comparison tasks. Finally, we have investigated the association between nonsymbolic and symbolic Weber fractions and arithmetic, separately for MD and TA children. If nonsymbolic numerical magnitude processing is crucial for mathematics learning, performance in the nonsymbolic numerical magnitude comparison task may be strongly associated with arithmetic in both groups.

## METHODS

### Participants

We recruited students from the second to seventh grade of public and private schools of Belo Horizonte and Mariana, Minas Gerais-Brazil, to voluntarily participate in this study. We screened 270 students and invited 222 children with TA (scores above the 25^th^ percentile in the school achievement test) and MD (scores under the 25^th^ percentile in the mathematics subtest of the school achievement test) to complete an individual assessment. After the individual assessment, we excluded 26 children who had a poor performance in our numerical magnitude comparison tasks (an R^2^<0.20, a nonsymbolic weber fraction>0.60, or a symbolic weber fraction>0.80).

Our final sample was composed by 159 children in the TA group and 37 in the MD group. As shown in Table 1, groups matched in age and sex. While both groups had normal intelligence, the TA group had higher general intelligence scores when compared to the MD group.

**Figure 1.**
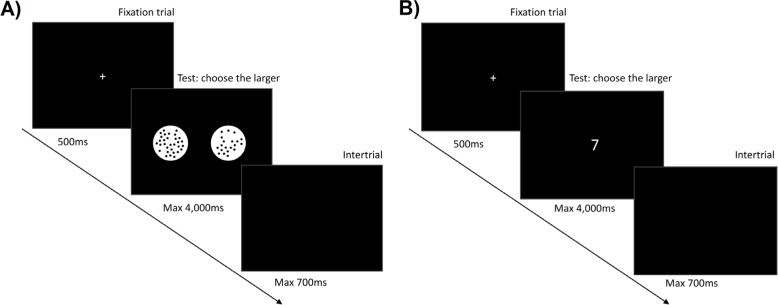
Illustration of experimental tasks, with arrows indicating the time curse of the tasks: fixation trial, experimental trial, and intertrial. (A) Nonsymbolic numerical magnitude comparison task and (B) symbolic numerical magnitude comparison task.

**Table 1.** Participants’ descriptive data.

	TA (n=159)		MD (n=37)		χ^2^	df	p-value	φ
Sex (% female)	58.49		48.64		0.82	1	0.36	0.07

	Mean	SD	Mean	SD	t	df	p-value	d
Age (years)	9.74	1.13	9.81	1.20	0.33	194	0.74	0.001
Raven (Z score)	0.51	0.62	0.08	0.74	3.66	194	<0.001	0.064

TA: children with typical achievement; MD: children with mathematics difficulties; Z score (mean=0, SD=1).

### Materials

#### Raven’s colored progressive matrices

The Raven’s Colored Progressive Matrices test was used to assess the general intelligence of participants. We calculated Z scores for each participant according to the Brazilian norms.^28^ In this test, children were instructed to find the best option to complete a pattern with a missing part.

#### The Brazilian school achievement test

The Brazilian school achievement test (TDE)^29^ has been the most widely used standardized test of school achievement with norms for the Brazilian population.^30^ This test comprises three subtests — mathematics, spelling, and reading — and is appropriate for students from second to seventh grades. In this study, the mathematics and the spelling subtests were used. The mathematics subtest is composed of three verbally presented word problems and 35 written arithmetic operations of increasing complexity. The spelling subtest is composed by 36 items. In the first item, children write their names, whereas in the other items they write a single-word orally presented. These subtests were used to characterize our sample’s school achievement and identify groups with mathematics difficulties (percentile in the mathematics subtest <25^th^).

#### Nonsymbolic numerical magnitude comparison

In the nonsymbolic numerical magnitude comparison task (Figure 1A),^15^ participants were instructed to compare two sets of dots presented simultaneously, and to choose the larger numerosity by pressing a key congruent to its side (left or right). Black dots were presented on a white circle over a black background. On each trial, one of the two white circles had 32 dots (reference numerosity) and the other one had 20, 23, 26, 29, 35, 38, 41, or 44 dots. Each numerosity was presented 8 times, with a total of 64 testing trials. Maximum stimulus presentation time was 4,000 ms, and intertrial interval was 700 ms. Between each trial, a fixation point appeared on the screen for 500 ms. To prevent the use of non-numerical cues, the sets of dots were designed and generated using a predefined MATLAB script,^31^ in such a way that, in half of the trials, the dots’ size remained constant and the total dot area covaried positively with numerosity. In the other half trials, the total dot area was fixed and the dots’ size covaried negatively with numerosity. Data were trimmed in two interactive steps for each child to exclude responses±3 SD from their individual mean reaction time (RT). As a measure of the ANS acuity, the Weber fraction (w_nonsymbolic_) was calculated for each child based on the Log-Gaussian model of number representation,^32^ with the methods described by Piazza et al.^17^

#### Symbolic numerical magnitude comparison

In the symbolic numerical magnitude comparison task (Figure 1B),^27^ children were instructed to judge if an Arabic number presented on the computer screen was greater or smaller than 5. The numbers presented on the screen were 1, 2, 3, 4, 6, 7, 8, or 9, printed in white over a black background. If the presented number was smaller than 5, children should press a predefined key on the left side of keyboard. Otherwise, if the presented number was greater than 5, children should press a key on the right side of the keyboard. The task comprised a total of 80 trials, 10 trials for each numerosity. The presented number was shown on the screen for 4,000 ms, and the time interval between trials was 700 ms. Before the test trial, there was a fixation trial with duration of 500 ms. As in the nonsymbolic numerical magnitude comparison task, responses±3 SD from individual mean RT were excluded. As an index of participants’ performance in this task, we calculated a symbolic Weber fraction (w_symbolic_), based on the Log-Gaussian model of number representation.^32^ As traditionally done in nonsymbolic numerical magnitude comparisons, the error rates for each ratio were computed, and the Weber fraction was calculated using the methods proposed by Piazza et al.^27^

#### Basic arithmetic operations

The Basic Arithmetic Operation task^15^ consisted of addition (27 items), subtraction (27 items), and multiplication (28 items) operations, which were printed on separate sheets of paper. Arithmetic operations were organized in two blocks of increasing complexity. Tie problems were not used for addition and multiplication, and no negative results were included in the subtraction problems. Time limit per block was set in 1 min, and children were instructed to work as fast and accurate as they could. In this task, one point was given for each correct problem. We used the total scores in the addition, subtraction, and multiplication subtasks in our analysis.

#### Procedures

This study had approval from the local IRB (ETIC-42/08). Children took part in the study only after they gave oral assent, and their parents or legal guardians signed the consent form. Children’s assessment occurred in quiet and comfortable rooms in their schools. First, children completed the Raven’s Colored Progressive Matrices and the TDE in groups of five participants maximum. Later, children individually completed the nonsymbolic numerical magnitude comparison task, the symbolic numerical magnitude comparison task, and the basic arithmetic operations task in a counterbalanced order.

## RESULTS

To investigate group differences in nonsymbolic and symbolic numerical magnitude processing and arithmetic performance, we compared TA and MD children’s performance using the Student’s *t*-test. We have also investigated how nonsymbolic and symbolic numerical magnitude processing is associated with an arithmetic performance by conducting zero-order correlations and a series of regression models.

### Nonsymbolic and symbolic numerical magnitude processing

To investigate differences between TA and MD groups in numerical magnitude processing, we compared groups’ w_nonsymbolic_ and w_symbolic_. Intelligence was not included as covariate in our comparison analysis, since there was no significant correlation between Raven’s Z scores and either w_nonsymbolic_ (r=-0.07, p=0.33) or w_symbolic_ (r=0.03, p=0.69).

**Figure 2.**
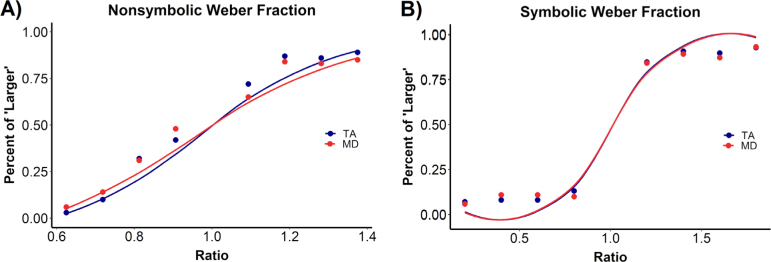
The weber fraction of children with typical achievement (TA, in blue) and mathematics difficulties (MD, in red) in (A) the nonsymbolic numerical magnitude comparison task and (B) the symbolic numerical magnitude comparison task. There was a significant group difference in nonsymbolic weber fraction (i.e., lower performance in the MD group). However, groups did not differ in the symbolic weber fraction.

Results indicated that children with MD had lower performance in the nonsymbolic numerical magnitude comparison task than children with TA, as shown in Figure 2A. Children with MD had higher w_nonsymbolic_ (M=0.31, SD=0.08) than their TA peers (M=0.26, SD=0.10), t(194)=2.94, p<0.01, d=0.52. In contrast, we observed no group differences in symbolic numerical magnitude comparison. MD children’s w_symbolic_ (M=0.22, SD=0.15) was not significantly different than TA’s w_symbolic_ (M=0.19, SD=0.12), t(194)=1.30, p=0.19, d=0.24.

### Association between nonsymbolic and symbolic numerical magnitude processing and arithmetic

First, we compared groups’ scores in the Basic Arithmetic Operations task. These results are presented in Table 2. Confirming the typical profile of arithmetic difficulties in MD, children with MD had lower performance than their TA peers in addition, subtraction, and multiplication.

**Table 2.** Participants’ performance in the Basic Arithmetic Operations task.

	TA (n=159)		MD (n=37)		Student’s t-test	df	p-value	d
	Mean	SD	Mean	SD				
Addition	22.19	4.57	18.30	6.03	4.38	194	<0.001	0.80
Subtraction	16.56	5.99	11.16	6.00	4.94	194	<0.001	0.91
Multiplication	14.62	8.51	7.51	6.96	4.73	194	<0.001	0.87

TA: children with typical achievement; MD: children with mathematics difficulties.

**Figure 3.**
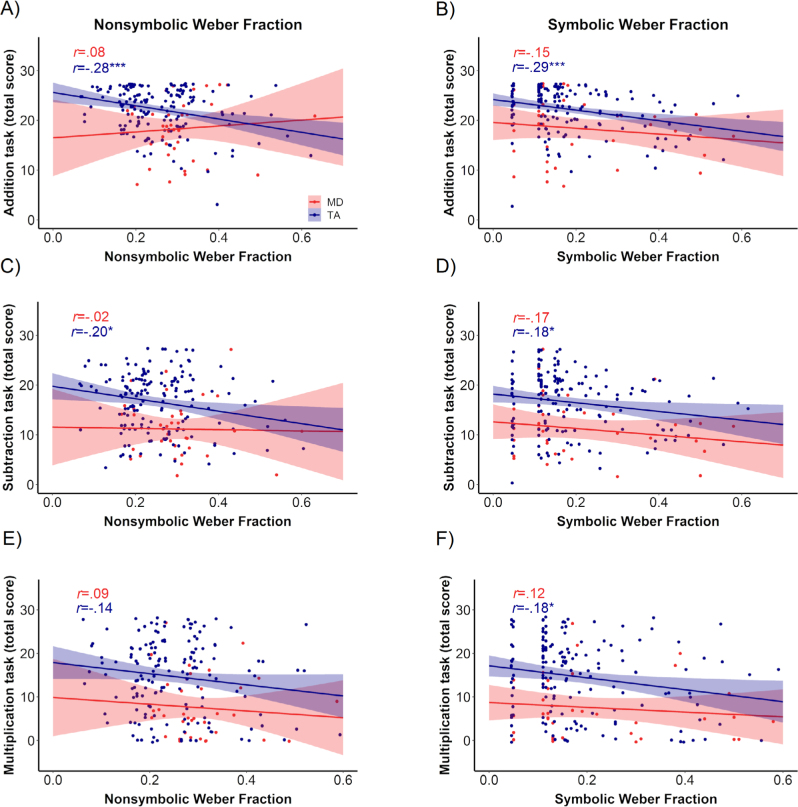
Association between numerical magnitude processing and arithmetics in children with typical achievement (TA, in blue) and mathematics difficulties (MD, in red). (A) correlation between nonsymbolic weber fraction and addition scores, (B) correlation between symbolic weber fraction and addition scores, (C) correlation between nonsymbolic weber fraction and subtraction scores, (D) correlation between symbolic weber fraction and subtraction scores, (E) correlation between nonsymbolic weber fraction and multiplication scores, and (F) correlation between symbolic weber fraction and multiplication scores.

To investigate the association between nonsymbolic and symbolic numerical magnitude processing and arithmetic in TA and MD, we ran zero-order correlations. As shown in Figure 3, in TA children, higher w_nonsymbolic_ was associated with lower performance in addition and subtraction. Furthermore, in this group, higher w_symbolic_ was associated with lower performance in addition, subtraction, and multiplication. In contrast, there was no significant correlation between either w_nonsymbolic_ or w_symbolic_ and arithmetic in children with MD.

R: Pearson’s correlation index; *p<0.05; ***p<0.001.

To investigate the specific contributions of nonsymbolic and symbolic numerical magnitude processing on arithmetic, we ran a series of regression models separately for each group. In the regression models, we included Raven’s Z scores, w_nonsymbolic_, and w_symbolic_ as factors and addition, subtraction, and multiplication scores as outcome variables. Our results for each arithmetic operation are presented by group in Table 3. In summary, results indicate that, in TA, nonsymbolic numerical magnitude processing significantly predicts performance in addition and subtraction. Furthermore, symbolic numerical magnitude processing significantly predicts addition and marginally predicts multiplication. In contrast, as we had observed in the zero-order correlation analyses, neither nonsymbolic or symbolic numerical magnitude processing predicted addition, subtraction, or multiplication in MD.

**Table 3.** Regression models.

Student’s t-test	TA (n=159)				MD (n=37)			
	β	Std. Error	t	p-value	β	Std. Error	Student’s t-test	p-value
Addition								
Raven (Z score)	−0.54	0.55	−0.99	0.32	−1.71	1.45	−1.18	0.25
w_nonsymbolic_	−10.35	3.71	−2.79	<0.01	2.19	12.37	0.17	0.86
w_symbolic_	−8.59	2.84	−3.02	<0.01	−4.03	6.72	−0.60	0.55
Subtraction								
Raven (Z score)	0.21	0.75	0.28	0.77	−1.78	1.44	−1.23	0.23
w_nonsymbolic_	−10.31	5.08	−2.03	<0.05	−5.17	12.29	−0.42	0.68
w_symbolic_	−6.70	3.88	−1.77	0.09	−4.78	6.68	−0.71	0.48
Multiplication								
Raven (Z score)	−0.48	1.08	−0.44	0.66	−3.32	1.62	−2.06	<0.05
w_nonsymbolic_	−8.99	7.26	−1.24	0.23	−15.12	13.79	−1.10	0.28
w_symbolic_	−10.85	5.56	−1.95	0.05	−1.87	7.49	−0.25	0.80

TA: children with typical achievement; MD: children with mathematics difficulties.

## DISCUSSION

In the present study, we investigated the performance of children with MD and TA in nonsymbolic and symbolic numerical magnitude comparison tasks. Furthermore, we investigated how performance in these tasks was associated with arithmetic. Results indicated that children with MD had worse performance (i.e., higher Weber fraction) in the nonsymbolic numerical magnitude comparison task than children with TA. However, we observed no group difference in the symbolic numerical magnitude comparison task. Finally, we observed that, in children with TA, performance in the nonsymbolic numerical magnitude comparison task was associated with addition and subtraction, and performance in the symbolic numerical magnitude comparison task was associated with addition, subtraction, and multiplication. In contrast, in children with MD, neither performance in the nonsymbolic or the symbolic numerical magnitude comparison task was associated with arithmetic. In summary, these results indicate that children with MD have difficulties in nonsymbolic numerical magnitude processing and do not engage basic nonsymbolic or symbolic numerical magnitude representations to solve arithmetic problems.

In our study, children with MD had lower acuity in nonsymbolic numerical magnitude processing than their TA peers. These results corroborate a pattern that has been found by most studies that used the Weber fraction as an index of performance.^10,13,15,20,21^ Therefore, the Weber fraction may be more sensitive to index nonsymbolic numerical magnitude processing than RT and accuracy alone.^10,33,34^

As symbolic numerical magnitude processing arouses nonsymbolic numerical magnitudes^9^ and relies on the ratio between numbers,^35,36^ it is plausible to use the Weber fraction as an index of performance in a symbolic numerical magnitude comparison task. We calculated a symbolic Weber fraction based on the Log-Gaussian model^32^ with methods adapted from Piazza et al.^17^ Using this index, we observed no difference between MD and TA groups. These results corroborate previous studies that found no evidence that children with MD have deficits in symbolic numerical magnitude processing, in particular in tasks using single-digit numbers.^14,26,37^

Regarding arithmetic operations, children with MD had difficulties solving addition, subtraction, and multiplication problems, in line with previous findings.^3,4,5^ In our study, both symbolic and nonsymbolic Weber fractions were associated with achievement in basic arithmetic operations in children with TA, but not in children MD. Corroborating results found previously by Pinheiro-Chagas et al.,^15^ these results indicate that children with MD do not engage their basic numerical magnitude representations to solve arithmetic operations. In contrast, they may use other strategies, such as counting their fingers, drawing sticks, or even guessing.^6^

Altogether, our findings corroborate the hypothesis that the main deficit in MD is processing nonsymbolic numerical magnitudes.^10^ This hypothesis proposes that children with MD have an impairment in their nonsymbolic numerical representations, which may lead to difficulties with symbolic numerical magnitudes and arithmetic tasks.^10,15^ Given their impaired numerical magnitude representations, children with MD may be more successful to solve arithmetic when these representations are not used, and other strategies are engaged.^15^ Deficits in the primitive, approximate nonsymbolic representations of numerical magnitudes may have a cascade effect across development, leading to the typical profile of arithmetic difficulties observed in MD.^3,10,13^

It is important to note that this study does not come without limitations. It is possible that the symbolic numerical magnitude task we used was not sensible enough to detect group differences, given its numerosity range (i.e., single-digits from 1 to 9). We decided to adopt this design since it has been extensively used in the literature.^3,14,22,23,37^ However, as Skagerlund and Träff^38^ noted, children are extensively presented with single-digit numbers in school, and multi-digit number tasks may be more sensitive to assess symbolic numerical magnitude processing. Nonsymbolic and symbolic magnitude comparison tasks with paired numerosity range should be used to investigate this subject, and our research group is working to clarify this in further studies. Furthermore, recent studies have shown that executive functions, particularly inhibition, highly influence performance in nonsymbolic numerical magnitude comparison tasks.^39,40^ Therefore, future studies should investigate nonsymbolic numerical magnitude processing in MD controlling for domain-general factors.

In this study, we investigated the nonsymbolic and symbolic numerical magnitude processing in children with MD and TA and how these types of magnitude processing are associated with arithmetics performance. Our results indicated that children with MD have deficits in nonsymbolic numerical magnitude processing. Furthermore, our results suggest that children with MD do not engage numerical magnitude representations to solve addition, subtraction, and multiplication tasks, which may explain their arithmetic difficulties. Number processing and arithmetic skills are important to academic performance, job stability, and mental health, in particular as our society increasingly values and demands the knowledge and application of science, technology, engineering, and mathematics (STEM).^1,2^ Therefore, it is important to identify underlying deficits in MD in order to establish better diagnosis criteria and consequently develop successful interventions.

## References

[1] Parsons S, Bynner J (2005). Does numeracy matter more?.

[2] Ritchie SJ, Bates TC (2013). Enduring links from childhood mathematics and reading achievement to adult socioeconomic status. Psychol Sci..

[3] Landerl K, Bevan A, Butterworth B (2004). Developmental dyscalculia and basic numerical capacities:a study of 8-9-year-old students. Cognition..

[4] Berteletti I, Prado J, Booth JR (2014). Children with mathematical learning disability fail in recruiting verbal and numerical brain regions when solving simple multiplication problems. Cortex..

[5] Ostad SA (1999). Developmental progression of subtraction strategies: A comparison of mathematically normal and mathematically disabled children. Eur J Spec Needs Educ..

[6] Geary DC (1993). Mathematical disabilities: cognitive, neuropsychological, and genetic components. Psychol Bull..

[7] Hanich LB, Jordan NC, Kaplan D, Dick J (2001). Performance across different areas of mathematical cognition in children with learning difficulties. J Educ Psychol..

[8] Siegler RS, Robinson M (1982). The development of numerical understandings. Adv Child Dev Behav..

[9] Dehaene S (1992). Varieties of numerical abilities. Cognition..

[10] Piazza M, Facoetti A, Trussardi AN, Berteletti I, Conte S, Lucangeli D, Dehaene S (2010). Developmental trajectory of number acuity reveals a severe impairment in developmental dyscalculia. Cognition..

[11] Noël MP, Rousselle L (2011 Dec 21). Developmental changes in the profiles of dyscalculia: an explanation based on a double exact-and-approximate number representation model. Front Hum Neurosci..

[12] De Smedt B, Noël MP, Gilmore C, Ansari D (2013). How do symbolic and non-symbolic numerical magnitude processing skills relate to individual differences in children's mathematical skills? A review of evidence from brain and behavior. Trends Neurosci Educ..

[13] Mazzocco MMM, Feigenson L, Halberda J (2011). Impaired acuity of the approximate number system underlies mathematical learning disability (Dyscalculia). Child Dev..

[14] Landerl K, Fussenegger B, Moll K, Willburger E (2009). Dyslexia and dyscalculia: two learning disorders with different cognitive profiles. J Exp Child Psychol..

[15] Pinheiro-Chagas P, Wood G, Knops A, Krinzinger H, Lonnemann J, Starling-Alves I (2014). In how many ways is the approximate number system associated with exact calculation?. PloS One..

[16] Price GR, Holloway ID, Rasanen P, Vesterinen M, Ansari D (2007). Impaired parietal magnitude processing in developmental dyscalculia. Curr Biol..

[17] Piazza M, Izard V, Pinel P, LeBihan D, Dehaene S (2004). Tuning curves for approximate numerosity in the human parietal cortex. Neuron..

[18] Chesney D (2016). The relationship between the numerical distance effect and approximate number system acuity is non-linear.

[19] Halberda J, Mazzocco MMM, Feigenson L (2008). Individual differences in non-verbal number acuity correlate with Maths achievement. Nature..

[20] Costa AJ, Silva JBL, Chagas PP, Krinzinger H, Lonneman J, Willmes K, Wood G, Haase VG (2011). A hand full of numbers: a role for offloading in arithmetics learning?. Front Psychol..

[21] Olsson L, Östergren R, Träff U (2016). Developmental dyscalculia: A deficit in the approximate number system or an access deficit?. Cog Dev..

[22] Rousselle L, Noël MP (2007 Mar). Basic numerical skills in children with mathematics learning disabilities:a comparison of symbolic vs non-symbolic number magnitude processing. Cognition..

[23] Iuculano T, Tang J, Hall CWB, Butterworth B (2008). Core information processing deficits in developmental dyscalculia and low numeracy. Dev Sci..

[24] Kovas Y, Giampietro V, Viding E, Ng V, Brammer M, Barker GJ (2009). Brain correlates of non-symbolic numerosity estimation in low and high mathematical ability children. PLoS One..

[25] Wong TT, Ho CS, Tang J (2017). Defective number sense or impaired access? Differential impairments in different subgroups of children with mathematics difficulties. J Learn Disabil..

[26] Landerl K, Kölle C (2009). Typical and atypical development of basic numerical skills in elementary school. J Exp Child Psychol..

[27] Oliveira-Ferreira F, Wood G, Pinheiro-Chagas P, Lonnemann J, Krinzinger H, Willmes K (2012). Explaining school mathematics performance from symbolic and nonsymbolic magnitude processing:similarities and differences between typical and low-achieving children. Psychol Neurosci..

[28] Angelini AL, Alves ICB, Custódio EM, Duarte WF, Duarte JLM (1999). Matrizes progressivas coloridas de Raven – escala especial.

[29] Stein LM (1994). TDE – Teste do Desempenho Escolar. Manual para aplicação e interpretação.

[30] Oliveira-Ferreira F, Costa DS, Micheli LR, Oliveira LDFS, Pinheiro-Chagas P, Haase VG (2012). School Achievement Test: Normative data for a representative sample of elementary school children. Psychol Neurosci..

[31] Dehaene S, Izard V, Piazza M (2005). Control over non-numerical parameters in numerosity experiments.

[32] Dehaene S, Haggard P, Rossetti Y, Kawato M (2007). Sensorimotor Foundations of Higher Cognition: Attention and Performance.

[33] Schneider M, Beeres K, Coban L, Merz S, Susan SS, Stricker J (2017). Associations of non-symbolic and symbolic numerical magnitude processing with mathematical competence: a meta-analysis. Dev Sci..

[34] Júlio-Costa A, Starling-Alves I, Lopes-Silva JB, Wood G, Haase VG (2015). Stable measures of number sense accuracy in math learning disability:Is it time to proceed from basic science to clinical application?. Psych J..

[35] Moyer RS, Landauer TK (1967). Time required for judgments of numerical inequality. Nature..

[36] Reynvoet B, De Smedt B, Van den Bussche E (2009). Children's representation of symbolic magnitude: the development of the priming distance effect. J Exp Child Psychol..

[37] Mussolin C, Mejias S, Noël MP (2010). Symbolic and nonsymbolic number comparison in children with and without dyscalculia. Cognition..

[38] Skagerlund K, Träff U (2014). Development of magnitude processing in children with developmental dyscalculia: space, time, and number. Front Psychol..

[39] Gilmore C, Attridge N, Clayton S, Cragg L, Johnson S, Marlow N, Simms V, Inglis M (2013). Individual differences in inhibitory control, not non-verbal number acuity, correlate with mathematics achievement. PLoS One..

[40] Bugden S, Ansari D (2016). Probing the nature of deficits in the 'approximate number system'in children with persistent developmental dyscalculia. Dev Sci..

